# Tolerance Induction in Vascularized Composite Allotransplantation—A Brief Review of Preclinical Models

**DOI:** 10.3389/ti.2023.10955

**Published:** 2023-02-09

**Authors:** Lioba Huelsboemer, Martin Kauke-Navarro, Stefan Reuter, Viola A. Stoegner, Jan Feldmann, Tobias Hirsch, Maximilian Kueckelhaus, Alexander Dermietzel

**Affiliations:** ^1^ Division of Plastic and Reconstructive Surgery, School of Medicine, Yale University, New Haven, CT, United States; ^2^ Institute of Musculoskeletal Medicine, University Hospital Münster, Münster, Germany; ^3^ Division of General Internal Medicine, Nephrology and Rheumatology, Department of Medicine D, University Hospital Münster, Münster, Germany; ^4^ Department of Plastic, Aesthetic, Hand and Reconstructive Surgery, Hannover Medical School, Hanover, Germany; ^5^ Division of Plastic Surgery, Department of Trauma, Hand and Reconstructive Surgery, Institute of Musculoskeletal Medicine, University Hospital Münster, Münster, Germany; ^6^ Department of Plastic, Reconstructive and Aesthetic Surgery, Hand Surgery, Fachklinik Hornheide, Münster, Germany

**Keywords:** Vascularized composite allotransplantation, tolerance induction, animal models, swine, rodent

## Abstract

Pre-clinical studies are an obligatory tool to develop and translate novel therapeutic strategies into clinical practice. Acute and chronic rejection mediated by the recipient’s immune system remains an important limiting factor for the (long-term) survival of vascularized composite allografts (VCA). Furthermore, high intensity immunosuppressive (IS) protocols are needed to mitigate the immediate and long-term effects of rejection. These IS regiments can have significant side-effects such as predisposing transplant recipients to infections, organ dysfunction and malignancies. To overcome these problems, tolerance induction has been proposed as one strategy to reduce the intensity of IS protocols and to thereby mitigate long-term effects of allograft rejection. In this review article, we provide an overview about animal models and strategies that have been used to induce tolerance. The induction of donor-specific tolerance was achieved in preclinical animal models and clinical translation may help improve short and long-term outcomes in VCAs in the future.

## Introduction

To date, about 150 vascularized composite allotransplants (VCAs) including over 40 face and 120 extremity transplantations, have been performed worldwide. Approximately 40 VCA programs across five continents have been established to date, and more VCA centers are anticipated to be established in the future (1,2).

In the field of reconstructive surgery, the geometric uniqueness and often resulting functional deficit of a composite tissue defect are major challenges. Vascularized composite allotransplantation (e.g., facial VCA) has revolutionized restoration of form and function of the most complex defects. For example, facial transplantation can now be offered to selected patients at experienced centers with reproducible results (3-5). However, acute and chronic rejection as well as the resulting need for life-long multidrug immunosuppression (IS) limit the more widespread use of this revolutionary biotechnology.

Side-effects related to these IS protocols (e.g., increased susceptibility to infection, malignancy, and organ dysfunction) continue to adversely affect the risk-benefit ratio transplants, particularly in case of VCA which is not a lifesaving but rather a life-giving procedure.

In this context, tolerance induction, the long-term immunosuppression-free graft acceptance without clinical or histological evidence of rejection, has become the topic of many preclinical and clinical research endeavors. The strategy of tolerance induction is often viewed as the holy grail of achieving improved transplant outcomes for example by preventing or slowing the development of chronic rejection, which is still an important long-term cause of graft loss in solid organ transplantation and presumably also in VCA (6,7).

Given the inherent complexity and diversity of VCA procedures, different animal models have been established and evaluated, while additional models are being developed to help answer specific VCA related immunological questions. We present a brief review of the most commonly used preclinical approaches of tolerance induction in the field of VCA.

## Tolerance Induction in VCA

**FIGURE 1:**
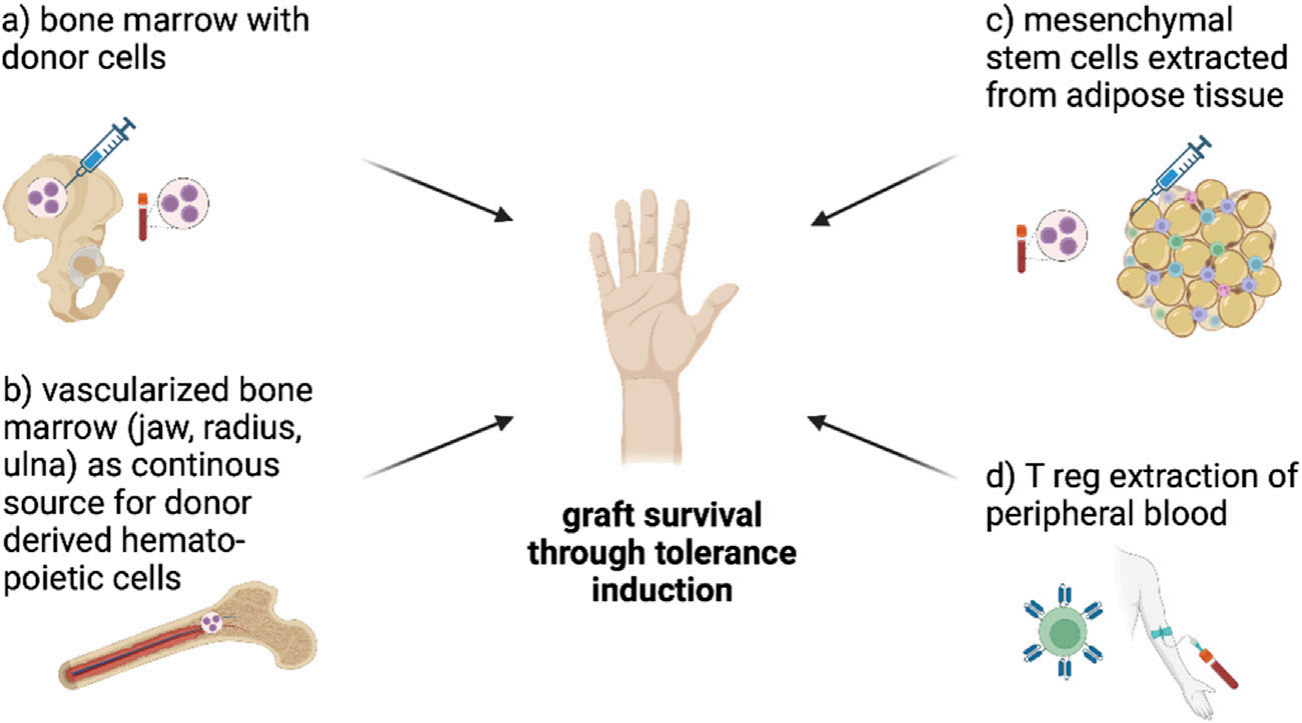
Figure shows cell-based tolerance induction approaches in VCA. Bone marrow transplantation with donor-derived stem cells can induce mixed chimerism and thereby induce tolerance **(A)**, vascularized bone marrow as continuous source for donor derived hematopoietic cells might be able to help minimize immunosuppressants **(B)** and mesenchymal stem cells extracted from adipose tissue **(C)** are already tested in VCA to induce tolerance through mixed chimerism and direct effects on the graft. The extraction of Treg cells **(D)**, modification into CAR-Tcells using CRISPR/C9 and infusion into humans is tested in clinical trials with SOT so far and seems to be a promising approach to induce tolerance in VCA. CAR-Tcells can recognize MHC-I on donor cells and block their interaction with the recipient´s immune system to prevent rejection (Figure created with BioRender.com).

Tolerance induction strategies can be broadly categorized into cell- and pharmaceutical based strategies. The goal is to achieve graft acceptance and minimize the need for less specific and highly toxic systemic immunosuppression such as tacrolimus and MMF. The interest in cell-based strategies has been increasing over the past 10 years. Cell-based strategies include the use of stem (e.g.,: bone marrow transplant-derived, mesenchymal stem cells) and/or immune cells (e.g.,: dendritic cells, T reg cells) plus usually a co-administration of a drug therapy regimen (8). Figure 1 illustrates standard tolerance induction strategies that have been used in preclinical models of VCA. The goal of stem cell-based therapies is the establishment of mixed chimerism which was described in detail in 1988 by Sykes and Sachs as a phenomenon of host and donor bone marrow-derived cell coexistance (9). For example, mixed chimerism can be induced by transfer of donor hematopoietic stem cells (HSCs) from bone marrow or cytokine-mobilized peripheral stem cells. Typically, the recipient is conditioned in order to control alloreactivity and generate space for donor HSCs engraftment. The co-existence of donor and recipients HSC is thought to promote donor-specific tolerance while maintaining immunocompetence (10,11).

Mixed chimerism alone was shown to not consistently achieve tolerance induction and thus Leonard et al. described different approaches achieving mixed chimerism combined with adjuvant cellular therapies leading to tolerance induction in pigs (3,10). The longest tolerance induction through establishment of mixed chimerism was demonstrated in a pig model by Leonard et al. (12,13) in 2015 (14) after performing VCA in stable mixed chimerism or concurrent with induction of mixed chimerism following hematopoietic stem cell transplantation. Both groups showed no signs of rejection up to 504 days after transplantation.

To achieve a continuous supply of donor derived bone marrow elements, Barth et al. further analyzed the role of vascularized bone in a heterotopic partial face transplantat model in NHPs (15). VCA containing vascularized bone not only led to prolonged, but rejection free graft survival (430 days) compared to VCA without vascularized bone (7 days). Schneeberger et al. developed the “Pittsburg Protocol” as an approach in humans following upper-extremity transplantation. Bone marrow cell-based treatment allowed a maintenance low-dose immunosuppression protocol compared to conventional protocols (16). However, long-term results have not been published and further studies are needed to evaluate the safety and reliability of this approach.

Conditioning of the recipient entails toxic side effects, thus approaches of bone marrow infusion-mediated immunomodulation without the necessity of conditioning are studied at the time (17). In this context other immune cells have been studied. Mesenchymal stem cells (MSCs) are multipotent stem cells that can differentiate into mesenchymal cell lineages. MSCs are derived from the bone marrow but can also be extracted from for example adipose tissue (= adipose derived MSCs) and have shown promising immunomodulatory effects by regulation of T-cell proliferation and inhibition of dendritic cell differentiation as they interact with the innate and adaptive immune system (18). Not only can MSCs induce mixed chimerism in the recipient, but they can also modulate cytokine expression in VCAs and hereby may be able to prolong graft survival(19). Promising results have been shown in small and large animal models, and clinical trials using mesenchymal stem cells have been established in solid organ transplants with promising results (8).

Ongoing clinical trials in humans evaluate the potential of dendritic cells as major regulator of the human immune system. An injection of immature dendritic cells was shown to have an immunosuppressant effect and to achieve inhibition of memory T cells (20). Another promising approach is the use regulatory T-cells. Tregs help maintain and regulate self-tolerance, antimicrobial resistance, tumor immunity and transplant rejection. Tregs exert their immunosuppressive effects *via* cell-to-cell interaction with target immune cells, *via* removal of IL-2 (potent factor in T cell survival and growth), *via* anti-inflammatory molecules (TGF-beta) or through costimulatory pathways such as binding to cytotoxic T-lymphocyte-associated protein 4. Nevertheless, irregularity in Tregs can lead to autoimmune disorders, reduced disease tolerance or higher risk for cancer(21). Recent studies have investigated the potential therapeutic value of genetically modified patient-derived Tregs, such as antigen-specific Chimeric Antigen Receptor (CAR)-Tregs, targeting for example MHC-class I expressed by donor cells. Tregs can be isolated from patient´s blood samples, cultured and expanded to produce for example CAR-Treg cells. Those conditioned T cells can be infused into the patient. Clinical trials did show the safety, efficacy, and proof of concept of Treg therapy in kidney and liver transplantation (22). The engineering of Tcells (e.g.: CAR-Tregs) might be a promising supplementary treatment of rejection in the future. CAR-T cells are typically designed to recognize donor MHC molecules, thus localizing to donor tissues and exerting their regulatory effects in a precise and targeted manner (21).

Pharmaceutical therapy regimens are the current gold standard in VCA and are mostly modifications of regimens that have already been established in SOT (23). Usually, an induction therapy (e.g.,: Anti-Thymoglobulin, Alemtuzumab) is administered first and a maintenance immunosuppressive regimen (Tacrolimus, Mycophenolate Mofetil (MMF), Prednisone) is given (8). Anti-Thymocyte globulin (ATG) is used as induction agent in most cases and leads to decreased T-cell mediated rejection (10). The maintaining therapy of the calcineurin inhibitor (CNI) Tacrolimus, Corticosteroids (Prednisone) and MMF (inhibits proliferation of lymphocytes) often involves side effects such as myopathy, diabetes mellitus, impaired kidney function, abdominal pain or diarrhea. Aside from the standard immunosuppressive regiments, other pharmaceutical approaches have been tested, for example tolerance inducing medications.

**FIGURE 2:**
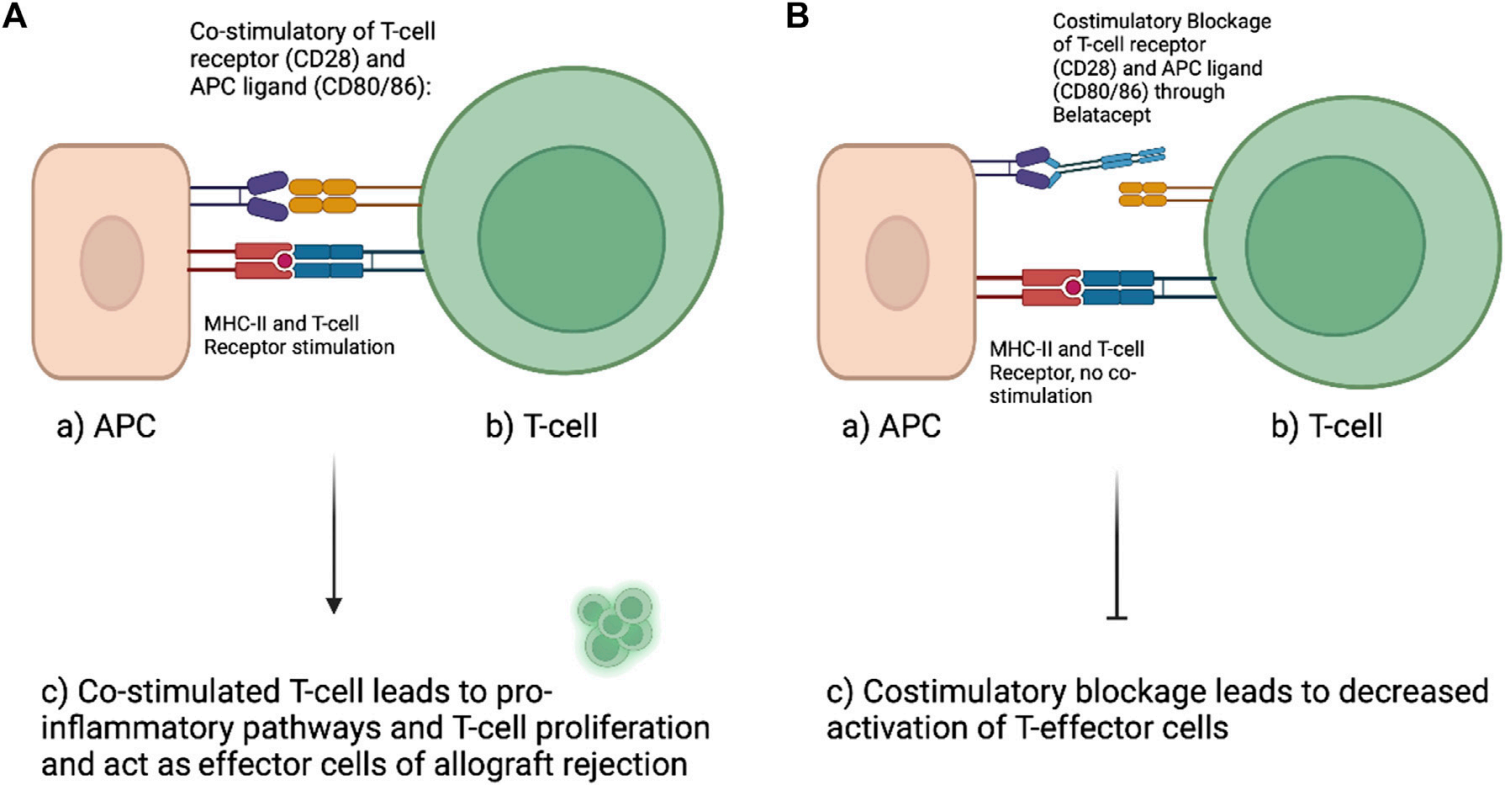
Left **(A)** demonstrates physiologic naïve T-cell activation. Antigen presenting cells (APC) interact with the CD28 surface receptor of naïve T-cells with their ligands CD80/86 while the antigen is presented to the T-cell. This interaction will promote T-cell proliferation and activation of cellular inflammatory pathways. **(B)** With the use of Belatacept (second-generation CTLA4-Ig) the costimulatory interaction between APC and T-cell can be altered. Belatacept blocks the interaction of CD28 with CD80/86 by binding to CD80/86. This interaction limits the activation of T-cells and was shown to reduce alloreactivity and thereby prolong allograft survival (Figure created with BioRender.com).

Cell mediated graft rejection in VCA is fueled by the CD28 and CD80/86 co-stimulatory pathway of T-cells (Figure 2). Preventing or interfering with co-stimulation of T cells is an approach for improved allograft survival and a more specific pharmaceutical approach that may cause less severe side effects compared to the conventional approaches (11). CD28 surface T-cell receptors communicate with antigen-presenting cells through CD80 and CD86 ligands and evokes both T-cell proliferation and pro-inflammatory pathways. Drugs like Belatacept or Abatacept are already established anti-rejection medications used in humans to interfere with co-stimulatory pathways. Both inhibit the costimulatory pathway through CTLA4-Ig or CD 125 (Fusion protein of cytotoxic T-lymphocyte-associated antigen 4 Immunoglobulin and IgG1 Fc) by inhibiting the linking of CD28 (T-cell surface) and CD80/86 (APC surface) (15,17). Belatacept can serve as replacement for CNI after kidney transplantation as side effects appear to be less than with CNI. Belatacept is already in clinical application following hand and/or face transplantation along with low dose of CNI/MMF/prednisone. Subsidiary, topical administration of immunosuppression might also reduce the systematically dosage of immunosuppressants. Tacrolimus and steroids are available as creams or mouthwashes as “transplant-targeted therapy” and show promising results in first approaches (8).

## Large Versus Small Animal Model

Rodent models are of great value especially in VCA research and are most popular for preclinical studies as they allow precise genetic breeding, are easy to reproduce and handle, produce low costs in housing and breeding and enable therefore larger study groups. The majority of the described animal models in this article used rat models and most commonly the hindlimb model was used. Mice allow more precise genetic manipulation which makes analysis of immunological mechanisms easier when compared to other small animal models. However, disadvantages of rat and mice models are related to their smaller size in terms of anatomic structures and the lack of similarity to the human immune system. Furthermore, the results of rodent models have often failed to be successfully transferred to large animal models or human trials (8). In addition, their short lifespan can make it difficult to truly assess long-term graft survival. Most importantly, the development of a naive immune system in largely germ-free laboratory environments and the lack of class II antigen expression on rodent vascular endothelium suggests a fundamentally different immune system than that of humans (11, 24-27).

Large animals are suited as preclinical models as they allow evaluation of a hypothesis in a complex system that has strong similarities to humans in terms of anatomical structures, size, and immune responses, making data potentially more reliable for translation to humans (28). Swine models also allow genetic manipulation to control for example the degree of MHC mismatch in transplantation and share great similarity with humans in structure, cellular and antigenic attributes in skin (8). NHP models are also of great value as they are even more similar in human anatomy than pigs and showed similar transplant rejection to humans. Hand transplantation is feasible and did show good motor function in NHPs (29). Disadvantages are cost and for example with primates’ ethical concerns, slower reproductive cycles. Especially the skin of humans and pigs show a great similarity which is even more important with skin being a crucial part of VCA and complicating factor on the way to long-term graft survival and tolerance induction. Having a great similarity with pig skin makes pig animal model most valuable for research. Finally, the high costs of livestock breeding and research in large animals, the ethical status in NHP models on the one hand and the simplicity of reproducibility, handling of rodents and low costs on the other hand may explain why most research groups are using rodents.

### Mouse Models

Costimulatory Blockade (CoB) takes advantage of the mechanism that naïve helper and cytotoxic Tcells must be activated through different pathways to become effector T-cells that can ultimately promote graft rejection. Antibodies, antigens, and immunosuppressive drugs (e.g.: Belatacept) can inhibit T-cell costimulatory pathways and, combined with adjuvant strategies such as total body irradiation (TBI) or stem cell transplantation, may induce mixed chimerism and thereby induce graft tolerance in VCA (10). Tolerance induction in all mice models described here (Table 1) was achieved *via* drug-based strategies utilizing costimulatory blockade in combination with adjuvant stem cell and/or TBI therapy regimen. Tolerance has been successfully induced up to 120–210 days in all mouse models described in this article. Five out of six models used an osteomyocutaneous hindlimb allograft transplant model while one used a full-thickness skin graft model without inclusion of bone or muscles. Lin et al. (2016) and Anggelia et al. (2021) showed successful induction of donor-specific tolerance (survival >120 days) by application of CoB (anti-CD154, CTLA4-Ig) with a short treatment of rapamycin (30,31). Both studies achieved graft survival up to 120 days. Lin et al. (2021) added high-dose bone marrow transplantation cells to the regimen of CoB (anti-CD154, CTLA4-Ig) and rapamycin and induced tolerance by mixed chimerism for >120 days in vascular bone marrow transplantation (32). Oh *et al.* (2020) combined the CoB (anti-CD154, CTLA4-Ig) with TBI and demonstrated tolerance induction and a depletion of alloreactive T cells for >210 days. Depletion of alloreactive T cells appears to be a promising mechanism for long-term graft feasibility and a key to long-term viability in this approach (33). Lin et al. (2013) used a different approach *in vitro*; the group used a donor antigen-specific CD4^−^CD8^−^ double negative Treg-based therapy plus anti-lymphocyte serum (ALS) plus rapamycin plus Il-2/Fc fusion proteins and showed tolerance induction in VCA but interestingly not in full thickness grafts (34). Davis et al. (2014) applied adipose-derived stromal cells (ASC) with non-myeloablative low-dose busulfan plus anti-CD4/CD8 and induced tolerance for >180 days as well as mixed macrochimerism (35). The longest graft survival plus depletion of alloreactive Tcells for over 210 days could be shown *via* CoB plus TBI 1 day before surgery by Oh et al (2020). This approach is furthermore relinquishing immunosuppressants which could be a promising in terms of that patients are mostly young and healthy when receiving VCA and therefore suffer a lot from long-term toxic side effects.

**TABLE 1:** VCA models with detailed therapy regimen in Mice, all models developed tolerance induction.

Mouse: VCA model	Donor	Recipient	Therapy regimen	Days of survival	Results	Ref
Osteomyocutaneous VCA or full thickness skin (FTS) transplantation fully MHC mismatched	DBA/2	C57BL/6	Skin grafting each *n* = 3: I = ALS & Rapa (*n* = 3), II = ALS, Rapa & DN, IV = ALS, Rapa, IL-2 & DN	10–180	Antigen-induced CD4 derived DN Tregs and a short course of anti-lymphocyte serum, rapamycin and IL-2/Fc fusion protein results in tolerance in VCA but not FTS allografts	(34)
			VCA each *n* = 5: I = untreated, II = Rapa, III = Rapa & IL-2, IV = Rapa & DN, V= Rapa, IL-2 & DN, VI = ALS, Rapa & IL-2, VII = ALS, Rapa, IL-2 & DN			
Osteomyocutaneous allografts (OMC)	Balb/c (H2d)	C57BL/6 (H2b)	I = without Treg depletion (*n* = 15), all other groups received anti-CD154, CTLA4-Ig & Rapa: II = POD 0 (*n* = 7), III = POD 30 (*n* = 7), IV = POD 90 (*n* = 7)	50–180	80% of VCA receipients with CoB & Rapa developed tolerance, 20% showed signs of rejection	(31)
Full-thickness trunk skin grafts	Balb/c	C57BL/6	I = untreated control (*n* = 10), II = conditioning therapy only (*n* = 10), III = conditioned with skin transplantation received ASCs (*n* = 6), IV = conditioned with skin transplantation received ASCs & BMCs (*n* = 12) Conditioning: anti-CD4 & anti CD8 monoclonal Anti bodies and non-myeloablative low dose busulfan	17–58	BMCs &ASCs results in skin allograft survival and mixed donor-recipient macrochimerism	(35)
Osteomyocutaneous allografts (alloOMCs) or myocutaneous allografts (alloMC)	Balb/c (H2d)	C57BL/6 (H2b)	I = alloMC with 1.5 × 10^8 CBMT (*n* = 6), II = alloMC with 3 × 10^7 CBMT (*n* = 6), alloOMC with VBMT (*n* = 6), IV = syngeneic group OMC without treatment (*n* = 4), V = alloMC without CBMT (*n* = 6) All groups received a costimulated blockage: anti-CD154 & CTLA4Ig plus Rapa short-term	62–120	VBMT with CoB & Rapa led to prolonged graft survival (>120 days), high CBMT also led to prolonged graft survival	(32)
Orthotopic hindlimb transplantation and full thickness skin trans-plantation (third party) fully MHC mismatched	Balb/C (H2d) FVB/N (H2q)	C57BL/6 (H2b)	I = untreated (*n* = 5), II = CTLA4-Ig (*n* = 4), III = CTLA4-Ig & hamster anti-mouse CD154 mAB (*n* = 8), IV = TBI 1 day before surgery, CTLA4-Ig & hamster anti-mouse CD154 mAB (*n* = 6)	8–210	CoB treatment plus TBI 1 day before Surgery increased graft survival (82 days) and showed T cell depletion	(33)
Osteomyocutaneous allografts (alloOMCs) or myocutaneous allografts (alloMC)	Balb/c (H2d)	C57BL/6 (H2b)	I = syngeneic controll (*n* = 2), II = alloOMC untretead	12–120	Combined CoB and Rapa (Group VI) led to long-term allograft survival (>120 days, 12 out of 15 animals) and same treatment in alloMC (Group III) without vascularized BM showed reduced allograft survival (53 days + - 11.6)	(30)
			(*n* = 5), II = alloMC with anti-CD154, CTLA4Ig & RPM			
			(*n* = 6), IV = alloMC with antiCD154, CTLA4Ig (*n* = 6),			
			V = alloOMC with Rapa (*n* = 6), VI = alloOMC with anti			
			CD154, CTLA4Ig & Rapa (*n* = 15)			

DN, double negative Treg based therapy; ALS, anti-lymphocyte serum; Rapa, rapamycin; ASCs, adipose-derived stromal/stem cells; VBMT, Vascularized bone marrow transplantation; CBMT, conventional bone marrow transplantation; CoB, Costimulated Blockade; BMCs, bone marrow cells.

### Rat Models

Tolerance induction was achieved in five out of eight described rat models (Table 2), and stem cells were used in most approaches to obtain longer-term graft survival. In total, four studies used a hindlimb, two a skin flap, one abdominal wall with/without hindlimb and one group an osseomusculuocutanous sternum with/without thymus model. Ramirez et al. (2013) showed a successfully induced tolerance and peripheral chimerism using a regimen of cyclosporine A (CsA), ALS, and adipocyte-derived stem cells (36). Jindal et al. (2015) used human IL-2 fusion protein and combined this regimen with ALS plus CsA (37). This regimen showed the longest graft survival with >150 days plus tolerance induction but no mixed chimerism were analyzed.

**TABLE 2:** VCA models with detailed therapy regimen in Rats.

Rat: VCA model	Donor	Recipient	Therapy regimen	Days os survival	Results	Ref
Hindlimb, orthotopic	BN, RT1u	LEW, TR1l	Five groups received different circles of treatment *n* = 4 ALS, n = 5 CsA, *n* = 4 CsA + human (h) IL-2/Fc, *n* = 6 ALS + CsA, *n* = 5 ALS + CsA + hIL-2/Fc	8–150	hIL-2/Fc in combination with ALS + CsA enables long-term graft survival and induction of tolerance, furthermore hIL-2/Fc leads to increased Treg proliferation & function but no chimerism were analyzed	(37)
Groin flap fully MHC mismatched	ACI, RT1a for flap LEW for BMC	LEW, RT1I rec. flap	N = 8 each: I & II: VCA from ACI donors preconditioned at 24 & 72 hrs, prior to VCA transplant, supported with a 7-day IS protocol of s.c. CsA & i.p. anti-ab-TCR monoclonal antibody, III & IV: VCA from ACI donors precond. at 24 & 72 h, prior to transplantation (no IS). V: VCA from noncond. ACI donor under a 7-day IS of CsA & anti-ab-TCR. VI: VCA from noncond. ACI donor, no IS	8–98	The regimen BMC prior 24 h plus anti-ab-TCR/CsA shows the longest graft survival (80+-18 days) and tolerance induction with mixed chimerism	(41)
		ACI received BMC				
Osseomusculocutaneous sternum (OMCS, n = 5) or osseomusculocutaneous sternum and thymus (OMCST, n = 5) heterotopic allotransplantation	LEW BN, RT1l	LEW, RT1l	CsA monotherapy (16 mg/kg) tapered to 2 mg/kg and maintained for the duration of the study	150	Study confirms correlation between thymus transplantation and donor-specific chimerism. No signs of rejection in any of the transplants during the 150 days posttransplant (Banff grade 0) but also no tolerance induction	(43)
Heterotopic hindlimb fully MHC mismatched	BN, RT1An	LEW, RT1Al	I = syngeneic control (*n* = 4); II-VI (each *n* = 6) = allogeneic VCA with II = no treatment, III = ALG &CsA, IV = ALG, CsA & ADSC, V = ALG, CsA & TBI, VI = ALG, CsA, ADSC & TBI	10–150	150 days of survival in 4 out of 6 rats in group IV & VI, both received infusion of ADSC, tolerance induction was achieved here but no mixed chimerism were analyzed	(39)
Full-thickness hemiabdominal wall (HAW) or hindlimb osteomyocutaneous (HLOMC) or combined HAW/HLOMC flap fully MHC mismatched	BN, RT1An	LEW, RT1Al	I&II = syngeneic controls; III-V = no IS (III = HAW *n* = 6, IV = HAW/HLOMC *n* = 4, V=HLOMC *n* = 4); VI-VIII ALS, CsA & ADSC (VI = HAW *n* = 8, VII = HAW/HLOMV *n* = 7, VIII = HLOMC *n* = 8)	11–150	150 days of survival only in syngeneic control groups; longest survival in group VIII (HLOMC) with 57 +- 21 days, also showed significantly higher peripheral	(36)
					Chimerism as well as tolerance induction	
Groin flap fully MHC mismatched	ACI RT1A	LEW, RT1Al	*n* = 6 each group: I = untreated, II = 7 days anti-ab-TCR/CsA, III = DRCC, IV = DRCC plus 7 days anti-ab-TCR monoclonal antibody & CsA	8–125	Longest survival in group IV with 125 days after single application of DRCC plus IS regimen for 7 days. DRCC therapy induces tolerance induction plus peripheral blood chimerism in the VCA recipients	(40)
Inferior epigastric flap	BN, RT1An	LEW, RT1Al	*n* = 15 each group: I=VCA, II = VCA & hASCs/GFP, III = VCA & hASCs/CCR7	8–17	Targeted migration of hASCs/CCRP7 infusion to secondary lymphoid organs potently prolong the mean survival time of VCA flaps, no chimerism/tolerance was established	(42)
Hindlimb	BN, RT1An	LEW, RT1Al	I = control (*n* = 5), II = 1 × 10^6 BM-MSCs (*n* = 6), III = 5 × 10^6 BM MSCs (*n* = 7),IV = 1 × 10^6 AD-MSCs (*n* = 8), V = 5 × 10^6 AD-MSCs (*n* = 9) all recipients: rabbit antirat lymphocyte serum 4 days before & 1 day after surgery, daily Tacrolimus 0–21 days after surgery, single-shot MSC IV on day 1 after surgery	47–120	all cell-treated groups showed prolonged survival, 47% of the MSC-treated exhibited long-term survival of the allograft and tolerance induction (>120 days). Peripheral blood chimerism were only transient	(38)

ADSC, syngeneic adipose-derived stem cells of LEW rat; ALS, Antilymphocyte serum); anti-ab-TCR, anti-ab- T-cell receptor monoclonal antibody; ALG, anti-lymphocyte globulin; DRCC, donor-recipient chimeric cells intraosseous; CCR7, chemokine receptor 7; hASCs, human adipose-derived stem cells.

Plock et al. (2015) changed the regimen to only a short course of FK-506 plus either adipose- or bone marrow-derived mesenchymal stem cells and induced tolerance as well as long-term graft survival and transient mixed chimerism (>120 days) (38). Cheng et al. (2013) used adipocyte-derived stem cells, CsA and ALS. Tolerance induction and long-term graft survival could be achieved up to 150 days but no mixed chimerism were analyzed (39). A single donor-recipient chimeric cells (DRCC) therapy by *ex vivo* cell fusion (of bone marrow cells) was applied by Cwykiel et al. (2021) in combination with CsA and antibody therapy and resulted in graft survival of >79 days, tolerance induction plus peripheral chimerism (40). In 2016, Sieminow et al. showed establishment of mixed chimerism by preconditioning recipient bone marrow cells (BMC) followed by anti- α - β -T cell receptor (TCR) monoclonal antibody plus CsA for 7 days post-transplant and achieved a median graft survival of 90 days plus tolerance induction (41). Ma et al. (2019) demonstrated a potential attenuation of rejection by infusion of human adipose-derived stem cells (hASC) plus chemokine receptor 7 (CCR7). CCR7 was able to guide hASC to secondary lymphoid organs to have immunomodulatory effects on Tcells. Chimerism or tolerance induction have not been established (42). Zor et al. (2020) analyzed the role of a simultaneous thymus transplantation. Chimerism were detectable >150 days posttransplant under continuous CsA therapy in an osseomusculoutaneous sternum and thymus allotransplantation, but without induction of tolerance (43). The longest survival with simultaneously induction of tolerance here was shown by Cheng et al. (2013) with a regimen of adipose-derived stem cells, CsA and ALS with or without TBI. Unfortunately, the establishment of mixed chimerism has not been analyzed. Most approaches here show that using stem cell therapies can induce tolerance and graft survival.

### Swine Models

In swine (Table 3), tolerance induction was attempted by establishing mixed chimerism with and without co-stimulatory blockage. One group used a full-skin flap, the second group used an osteomyocutaneos flap. Leonard et al. (2014) successfully induced tolerance in their large animal model. The recipient was preconditioned by T-cell depletion plus TBI, followed by hematopoietic cell transplantation. VCA was either performed into stable mixed chimerism or after establishing of mixed chimerism 85–105 days later. After transplantation, a 45-day course of CsA was given. No signs of rejection were seen between 115–504 days posttransplant (14). Lellouch et al. (2022) showed tolerance induction by mixed chimerism across MHC class-I-mismatch up to 400 days posttransplant. Treatment was TBI and thymic irradiation 2 days before surgery. Bone marrow transplantation was performed on the day of surgery, followed by co-stimulatory blockade (CTLA4-Ig + FK405) for 30 days and treatment of anti-IL6R monoclonal antibody (mAb) plus methylprednisolone (44). Both approaches show promising results in terms of long-term graft survival, similarity to the human immune system and absence of long-term immunosuppression and serve as proof of concept in a preclinical large animal model.

**TABLE 3:** VCA models with detailed therapy regimen in Swine, both induced tolerance.

Swine: VCA model	Donor	Recipient	Therapy regimen	Days of survival	Results	Ref
Fasciocutaneous flap (MGH miniature swine)	SLAacPAA+	SLAadPAA-	T cell depletion with CD3-immunotoxin,	115–504	Both VCAs (transplanted into stable chimerism *n* = 4 and transplanted at time of hematopoietic cell transplantation (HCT) *n* = 2) showed no signs of rejection following withdrawl of immunosuppression and stable mixed chimerism	(14)
			100 cGy TBI prior to HCT, 45-day course of CsA			
Osteomyocutaneous flap matched for class II SLA, mismatched for class I SLA (MGH miniature swine)	PAA+	PAA-	300cGy total body irradiation +700cGy thymic irradiation, following bone marrow transplantation, co-stimulated blockade using CTLA4-Ig + FK506	400	3 out of 5 animals achieved long-term survival without Immunosuppression, 2 recipients developed idiopathic pneumonia-like syndrome (euthanized 36–39	(44)
					POD), tolerance induction and mixed chimerism was achieved	

CsA, Cyclosporine A.

### NHP Model

As described above, to have a continuous source for donor stem cells, vascularized bone marrow transplantation might be an approach to induce tolerance through establishment of mixed chimerism (continuous source of donor-derived hematopoietic stem cells). This approach was tested by Barth et al. (Table 4) as he further analyzed the role of vascularized bone marrow transplantation (VBM) in a VCA model with a heterotopic partial face transplantation in NHPs. VCA containing vascularized bone not only led to prolonged, but also rejection free graft survival (430 days) compared to VCA without vascularized bone (7 days). However, withdrawal of immunosuppressants led to graft loss and tolerance induction was not achieved but VBM enabled a low dose maintenance immunosuppressants regimen and sporadic macrochimerism were detected (15). Although this approach was unable to achieve tolerance induction through mixed chimerism, these results serve as proof of concept. However, stable macro or micro chimerism has not been documented in human VCA. Further studies are necessary, for example on how to enhance the ability of vascularized bone marrow to induce chimerism, e.g., *via* Treg modulative strategies to increase acceptance of donor derived hematopoietic stem cells (21).

**TABLE 4:** VCA model with detailed therapy regimen in Non-Human Primate (NHP); vascularized bone marrow (VBM), bone marrow cells (BMC) infusion.

NHP: VCA model	Donor	Recipient	Therapy regimen	Days of survival	Results	Ref
Heterotopic partial face transplantation with (*n* = 4)/without vascularized bone (*n* = 3) (mandible segment)	Cynomolgus macaques		Tacrolimus IV/IM plus MMF OV/orally and all daily	7–430	VCA with vascularized bone led to prolonged rejection-free graft survival and showed sporadic macrochimerism; no tolerance induction as graft was rejected after withdrawal but low dose maintenance of immunosuppression could be achieved	(15)
MHC class I-mismatched						

## Conclusion

Both small and large animal models are relevant for preclinical VCA research. Small animal models are advantageous because of low costs in breeding and housing and especially mice are well suited for analysis of immunological mechanisms. Large animal models offer greater similarity to human immune system and human anatomy, but models tend to be more complex and cost intensive. Tolerance induction in preclinical models was achieved using both cellular based and pharmaceutical based strategies. Clinical translation of such strategies to human trials has yet to be done successfully, however, the induction of donor-specific tolerance may ultimately help improve the risk benefit ratio of VCA transplantation.
